# Neuronal Circuit-Based Computer Modeling as a Phenotypic Strategy for CNS R&D

**DOI:** 10.3389/fnins.2019.00723

**Published:** 2019-07-16

**Authors:** Hugo Geerts, James E. Barrett

**Affiliations:** ^1^In Silico Biosciences, Inc., Berwyn, IL, United States; ^2^Center for Substance Abuse Research, Lewis Katz School of Medicine, Temple University, Philadelphia, PA, United States

**Keywords:** computer modeling, symptomatic treatment, psychiatry, disease modification, neurodegenerative diseases

## Abstract

With the success rate of drugs for CNS indications at an all-time low, new approaches are needed to turn the tide of failed clinical trials. This paper reviews the history of CNS drug Discovery over the last 60 years and proposes a new paradigm based on the lessons learned. The initial wave of successful therapeutics discovered using careful clinical observations was followed by an emphasis on a phenotypic target-agnostic approach, often leading to successful drugs with a rich pharmacology. The subsequent introduction of molecular biology and the focus on a target-driven strategy has largely dominated drug discovery efforts over the last 30 years, but has not increased the probability of success, because these highly selective molecules are unlikely to address the complex pathological phenotypes of most CNS disorders. In many cases, reliance on preclinical animal models has lacked robust translational power. We argue that Quantitative Systems Pharmacology (QSP), a mechanism-based computer model of biological processes informed by preclinical knowledge and enhanced by neuroimaging and clinical data could be a new powerful knowledge generator engine and paradigm for rational polypharmacy. Progress in the academic discipline of computational neurosciences, allows one to model the effect of pathology and therapeutic interventions on neuronal circuit firing activity that can relate to clinical phenotypes, driven by complex properties of specific brain region activation states. The model is validated by optimizing the correlation between relevant emergent properties of these neuronal circuits and historical clinical and imaging datasets. A rationally designed polypharmacy target profile will be discovered using reverse engineering and sensitivity analysis. Small molecules will be identified using a combination of Artificial Intelligence methods and computational modeling, tested subsequently in heterologous cellular systems with human targets. Animal models will be used to establish target engagement and for ADME-Tox, with the QSP approach complemented by *in vivo* preclinical models that can be further refined to increase predictive validity. The QSP platform can also mitigate the variability in clinical trials with the concept of virtual patients. Because the QSP platform integrates knowledge from a wide variety of sources in an actionable simulation, it offers the possibility of substantially improving the success rate of CNS R&D programs while, at the same time, reducing both cost and the number of animals.

## Introduction

The success rate of drug discovery projects in CNS disorders is at an all-time low with only single digit probability of success in clinical trials. The late-stage failure rate has been so prominent and costly, that many large pharmaceutical companies have abandoned neuroscience as a therapeutic area (Hyman, 2014a). Undoubtedly, these developments are due to a number of factors (Geerts, 2009) such as our current poor understanding of the pathophysiology underlying most of CNS disorders, posing significant hurdles to the development of appropropriate preclinical models in which to effectively translate findings into clinical efficacy. In addition, many drugs – especially antibodies – fail to engage the molecular target or, as will be discussed, target*s*, appropriately. CNS disorders are undoubtedly complex and the dissolution of the concept of the single target for these disorders has become increasingly evident.

**FIGURE 1:**
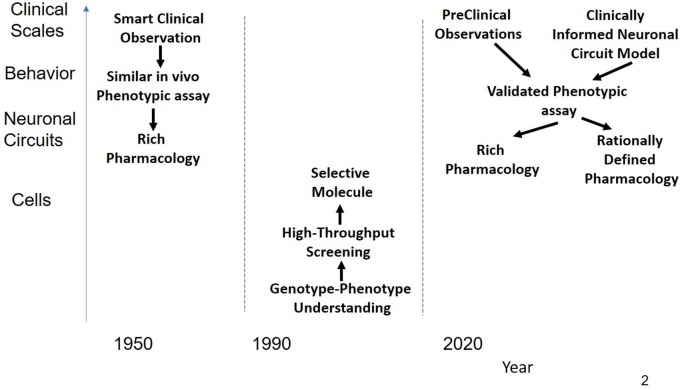
Schematic representation of the history of CNS R&D. Before 1990, drugs were discovered using a combination of phenotypic assays and smart clinical observation in the absence of any modern technological tools provided by molecular biology resulting in compounds with rich pharmacology that affected sufficient targets for a substantial clinical benefit. Often targets were found long after the molecules were on the market. From 1990 the emphasis shifted to rational single targets, based on insights and tools generated from molecular biology. Using high-throughput screening on cellular systems expressing these targets, very potent but highly selective drugs were identified. These drugs, however, did not shift the needle enough in the complex neuronal circuits to generate a clinical benefit. As a possible solution, novel CNS R&D strategies return to the basic principle of phenotypic assays either based on behavior observations in preclinical animal models or on models informed by neuronal circuits rather than single targets.

The majority of drugs that were introduced in the 1950s and 1960s for CNS indications such as schizophrenia and depression were successfully developed in a period without high-technological molecular tools (Figure 1) nor were the drug discovery efforts driven by a target-centric focus. The resulting compounds usually had a rich pharmacology, i.e., they interacted in with a multitude of targets, most of which were not even known at that time. A major figure of psychoactive drug discovery and development between 1955 and 1990 is Dr. Paul Janssen, founder of Janssen Pharmaceutica, who developed 75 successful drugs over the span of 40 years, with eight of them on the WHO List of Essential Medicines. We will review the strategy that was used to develop powerful and successful CNS medications during this time.

Molecular biology became a mature science from 1990 onward and generated a large number of powerful tools, such as deep sequencing, cloning of targets, and various sophisticated versions of transgene rodent models. Together with advanced imaging techniques with PET tracers and various MRI sequences., these developments have generated an enormous amount of information (“Big Data”) for which powerfull algorithms have been developed. The strategy underlying these approaches was based on the concept of “one gene, one protein, and one disease,” resulting in the identification of single targets that were supposed to be associated with a certain disease. Subsequent screening using high-throughput capabilities and powerful SAR driven medicinal chemistry resulted in highly potent and selective drugs. Unfortunately, there have been almost no new breakthrough drugs for CNS disorders discovered (Hyman, 2008, 2014b). This is particularly unfortunate for even the most widely used antidepressants successfully manage this condition in only 60% of the patients and there are really no effective drugs for the treatment of neurological disorders. For example, in Alzheimer’s Disease, the last 240 clinical development projects were all unsuccessful and the last approved medication – memantine – already dates from 2004 (Cummings et al., 2014). The relative success of rational target selection enjoyed in other indications such as oncology and inflammation has not occurred for CNS disorders.

In order to turn the tide of clinical trial failures, it is of interest to revisit the strategy that earlier drug hunters applied to develop successful drugs and to combine theses approaches with relatively recent developments to generate new insights and tools for CNS drug discovery. We propose an advanced computer modeling based phenotypic strategy that rests on our current understanding of how neuronal circuits drive human behavior, pharmacological mechanisms and data derived from clinical trials. This necessitates an integration of information derived from different disciplines, such as basic neurobiology, neuropharmacology, clinical data and neuroimaging, potentially leading to a much greater understanding of a polypharmacological profile with a greater positive impact on clinical outcome. We will give examples how this approach can help target validation, support rational multi-target drug discovery, even extrapolate findings from human induced pluripotent stem cells (hIPSC) to more elaborate neuronal circuitry and clinical candidate selection. In clinical development, this approach could help in identifying functional biomarkers for target engagement, optimal dose finding and quantifying the pharmacodynamic interaction with genotypes, disease state and co-medications.

## Phenotypically Driven Drug Discovery

The golden age of psychoactive drug discovery in the 1950s and 1960s witnessed the successful introduction of several CNS-active drugs, many of which are still widely used today or which have served as starting points for subsequent modifications. This relatively brief phase in the history of psychopharmacology was a remarkable period when novel and therapeutically effective drugs were introduced into clinical practice to treat schizophrenia, depression and anxiety. In none of these cases was there any mechanistic or pathophysiological understanding of these disorders. The identification of potential clinical utility was based on clinical observations of phenotypic changes that served as the basis for phenotypic drug screening and for the development of several animal models that attempted to capture clinical phenotypes with those in preclinical assays.

The discovery of haloperidol is a prime example of smart clinical observations with a phenotypical mind-set. Clinical observations on patients with schizophrenia noted that the symptoms were exacerbated when using drugs of abuse such as amphetamines. These observations led to the development of preclinical assays in rodents administered high doses of amphetamine producing some of the same heightened stereotyped behavior seen often in schizophrenic patients. As an early and insightful example of bedside-to-bench reasoning, this assay (amphetamine-induced stereotypy) is still being used to characterize new antipsychotics. On February 15, 1958, haloperidol (R1625) was synthesized in the Janssen labs and was found to block the effects of amphetamine on locomotor activity. It was approved by the FDA in 1967. Since then, more than 21,000 papers have been published on this drug which, without doubt, has been one of the most successful antipsychotic drugs.

It is important to note the chronology of this and related developments. Around the time of the synthesis of haloperidol, techniques for measuring dopamine were just being developed (Carlsson and Waldeck, 1958) and assays for the establishment of dopamine levels in the rodent brain became available the year after (Carlsson, 1959). Ironically, in the years following it’s discovery, haloperidol was used as a powerful tool to investigate the neurophysiology and neuropharmacology of the dopamine system *in vivo*. Binding of radio-active haloperidol allowed for the identification of a dopamine binding site in the mid-1970s (Snyder et al., 1975) that was followed eventually by the identification of this target after cloning of the D2 mRNA (Guennoun and Bloch, 1991). This approach also led to the development of an original method in the mid-1970s to study the effect of candidate drugs on other neurotransmitter systems in the absence of binding to cloned receptors. In the “ATN assay” (Niemegeers et al., 1977) behavioral effects were monitored after amphetamine (for the dopamine systems), tryptamine (5-HT system), and norepinephrine (NE). The different dose-responses for the challenges allowed to better dissect the interaction of multi-target pharmacology drug candidates.

Paul Janssen went on to develop many more antipsychotics with different profiles, some of them also acting on the serotonergic system (pimozide) and, therefore, could be classified as early atypical antipsychotics. Similar strategies were used in the field of analgesia and antifungals. It is without doubt that Janssen Pharmaceutica was a successful Drug Discovery and Development engine without having access to any of the current tools of molecular biology. So it is useful to explore the reasons for its success. Dr. Paul always talked about his 4 C’s : concept, concentration, commitment, and creativity.

Drug Discovery projects need to be concept-driven (biology-chemistry) rather than technology driven. Starting out with a very specific question, it is essential to identify the necessary techniques and tools – even if they are so basic, like pharmacology or enzymology. Avoid the “nice-to-have” and sexy technologies if they don’t contribute to the solution. Drug Discovery and Development has a different agenda than basic academic research. In addition, in order to address the complexities of the human brain, an integrative approach, rather than a reductionistic approach is essential for success.

A drug discovery project needs to be laser-sharp and concentrated on the end-goal which is to identify the best molecule for a given disease indication. In line with this, short feedback cycle times and early and continuous management buy-in are essential for success. Back in the days before e-mail, Webex and remote meetings due to the dispersion of research groups across different sites in pharma, Dr. Paul took the time to personally stop by in the labs to discuss the latest findings with the bench scientists. It was not unusual for Janssen scientists to be able to discuss the results of an interesting scientific finding with him on the same day. Besides the scientific feedback, it gave this approach a personal motivational push that allowed individuals to perform beyond what they thought were their limitations. A focused research organization is also not geographically dispersed. Almost all of the successful research at Janssen was performed on site in Beerse, Belgium.

Dr. Paul committed the necessary resources for a project; he believed research rhythms are different from economical, market, technology or grant cycles. Because he was so successful with his earlier products, he had the capability to think strategically. Long-term stability in the research environment is a major driver for success.

Like any biotech nowadays, creativity is a key driver of success; dare to go where nobody else has gone before. The Janssen laboratoria essentially created the market of antifungals and anti-helmintica, thanks to a number of veterinarians returning from the Belgian colony of Congo in the early 1960s. He also believed there is no basic or applied research; there is only good research and that the market is driven by quality drugs, not the other way around. By focusing on providing quality care for patients, benefits for other stakeholders will follow naturally.

Lastly, it cannot be emphasized too much that having a scientist as CEO was a major contributor to the success of a company (Leadership buy-in). That person needs to be able to see the challenges and opportunities of drug discovery beyond the mere numbers and metrics, and be driven by the motivation to make a difference for patients. In summary, many of these characteristics can be found in start-up biotech companies nowadays; however, the major difference was that Janssen Pharmaceutica at that time was a mature company with 17,000 employees in 30 countries.

## Rational Target Selection Driven Projects

### The Molecular Biology Revolution

From the early 1990s on, the number of molecular biology tools has steadily increased. Cloning and amplification of DNA started out in the early 1970s (Hershfield et al., 1974) and biotechnology was heralded as the next revolution in drug discovery and development 20 years later (Drews, 1995). The underlying premise was that genetic information would automatically “humanize” the R&D process and that therapeutic developments aimed at the cause of the disease rather than the symptoms could be developed. The strategy was to go from one gene, one protein to one disease. The overriding belief was that highly selective compounds would address the major underlying cause of the disease, would not engender some of the untoward side effects associated with “dirty drugs,” and would, therefore, provide powerful targeted therapeutic treatments.

Basically the idea was to fully deconstruct human biology to the level of genes and mutations and then build up the network of interacting genes and proteins, cell types and relevant circuits based on their interaction derived either from experimental data (such as yeast 2-hybrid) or from *in vitro* and *in vivo* experiments in rodents, leading to the concept of pathway analysis. At this juncture, however, the link with human physiology is often lost.

This approach has worked remarkably well for a number of diseases such as cardiovascular (see below for the story of PSCK9 inhibitors) or oncology, and for some rare diseases where the mutation in the gene identified the biological process driving the pathology. However, it became rapidly clear that no single mutation could explain the full pathology of the majority of (sporadic) CNS disorders. In most cases, such as in Alzheimer’s Disease, only a few families presented with dominant mutations, such as APP mutations and PS1/2 mutations in familial Early-onset Familial AD (Hardy, 1996) (less than 0.1% of cases).

There are a number of other major issues with this approach. First, it rapidly turned out that the findings for the familial cases are not readily generalizable to the sporadic form of the disease. For instance, a large number of therapeutic interventions aimed at removing beta-amyloid in AD have failed in the clinic and have led to questioning the amyloid hypothesis of this disease (Herrup, 2015; Karran and De Strooper, 2016). Second, for a number of genetic risk factors with high impact it has been very difficult to identify and characterize the relevant biology and to generate appropriate insights on the nature and properties of possible targets. For example, while the APOE gene was identified in Corder et al. (1993), it is still not clear what aspect of the biology drives the clinical phenotype and how to identify duggable targets (Lopez et al., 2014). Third, almost all proteins are subject to a myriad of posttranslational modifications which are often cell-dependent and dynamic in nature and are not reflected in the genetic or transcriptomic information.

### The Success Story of Genetically Driven Rational Drug Discovery

The poster child for a success story of genetically driven drug discovery and development is without doubt the development of proprotein convertase subtilisin kexin type 9 (PCSK9) inhibitors in cardiovascular diseases. Here the timeline from discovery of the gene to approval of two drugs is an amazingly short 12 years. Mutations in the PCSK9 gene that cause autosomal dominant hypercholesterolaemia were discovered in Abifadel et al. (2003) and 4 years later the crystal structure was determined (Piper et al., 2007). Clinical trial results for the first inhibitors evolucumab (Dias et al., 2012) and alirocumab (Stein et al., 2012) were published in 2012 and approval was granted by the FDA in 2015. Commercial success of the drugs has somewhat been limited due to the high price and the competition with generic cholesterol lowering drugs.

Rare diseases also benefit from genetic studies as the cause of the disease is very well known and often the pathway can be affected either by small molecules or other more advanced techniques such as oligonucleotides. These success stories include Kalydeco and Orkambi treatments for cystic fibrosis and the first ever FDA approval of the oligonucleotide Nusinersen for Spinal Muscular Atrophy.

### Highly Selective Drugs in CNS Disorders

Based on the observations that certain genes were involved in different aspects of schizophrenia, a number of highly selective drugs have been developed and subsequently tested in schizophrenia, notably to address the cognitive impairment and negative symptoms associated with schizophrenia. For instance, the finding that CHRNA7, the alpha7 nAChR gene, was associated with the clinical phenotype of schizophrenia (Leonard and Freedman, 2006), led to extensive clinical testing of a large number of alpha7 nAChR modulators (Geerts, 2012). Other attempts to develop highly selective drugs include PDE10 inhibitors (Geerts et al., 2016b), Histamine H3 antagonists, mGluR2/R3 partial agonists, dopamine D3, dopamine D4, glycine modulators, 5-HT2A modulators, GABA modulators, AMPAkines, and neurokinin modulators (for a review see Geerts and Kennis, 2014). At the present time, the clinical development of most of these targeted compounds has been halted due to lack of efficacy.

Similarly, in Alzheimer’s disease since the approval of memantine in 2004, 240 clinical trial projects have failed (Cummings et al., 2014). In this disease, the use of monoclonal antibodies for amyloid modulation is an extreme example of highly selective target selection. A likely reason for clinical trial failures is that highly selective compounds do not impact the general outcome of circuits sufficiently to lead to a robust clinical effect and that appropriately balanced effects on different pathways and circuits is essential.

However, we acknowledge that other aspects might play a role such as misaligned target engagement. For instance, all interventions aimed at glutamate and GABA are subject to a fine excitation-inhibition balance and often lead to an inverse U-shape dose-response. In the case of glycine modulation, basic neurophysiological processes such as the Hill properties for interaction of glycine with the co-agonist site on the NMDA receptor, together with the Na-Cl-Gly co-transporter system and the neuronal firing properties of pyramidal and inhibitory interneurons mandatory lead to an inverse U-shape dose-response (Spiros et al., 2014). This dose-response has indeed been confirmed in a PhII clinical trial with bitopertin (Umbricht et al., 2014) and probably was a major hurdle for successful confirmation in a Phase III study. Other reasons for the lackluster effect in CNS clinical development for highly selective compounds include wrong patient populations, the presence of a different co-morbidities, the formation of pharmacologically active metabolites, the impact of common genetic variants and mandatory clinical scales with a high subjective component. In addition to the underestimated impacts of gender (Podcasy and Epperson, 2016), large GWAS studies suggest that many genes each can contribute a very small amount to the pathology (Horwitz et al., 2019).

This raises the important question of patient subtypes and genetic heterogeneity in what appear to be common CNS disorders that might be defined as driven by a single gene, so that if only if we could identify that gene then a highly selective drug might be appropriate. However, with the exception of rare familial cases it is more likely that each different patient subpopulations might be driven by a unique and restricted set of low-impact genotypes.

A very important issue is the pharmacodynamic (PD) interactions between an investigative drug and other co-medications. Ironically, because of the rich pharmacology of approved CNS medications (see above), the probability of pharmacodynamic interaction with a highly selective drug is substantial, either direct (at the level of the receptor or target), but most importantly in an indirect way at the level of circuit outcomes. As an example, we studied the PD-PD interaction effects on cognitive impairment between antipsychotics, memantine, AChE-I such as donepezil, and galantamine and smoking in a virtual schizophrenia population (Geerts et al., 2015a). Each antipsychotic has a different interaction profile with these pro-cognitive interventions. For instance, olanzapine amplifies the effect of memantine in non-smokers; donepezil, but not galantamine, further enhanced the effect. Other antipsychotics such as risperidone, quetiapine, and aripiprazole had a negative interaction, while haloperidol was neutral. Most interactions became even more negative in smoking conditions. Such PD–PD interactions could explain apparently contradictory findings in clinical trials, but most importantly they can reduce the clinical signal in clinical trials if not addressed appropriately.

### Going Beyond Genomics

The scientific community quickly realized that genes or RNA sequences did not tell the whole story and new techniques were developed for documenting the changes in other more relevant readouts. From the late 1990s, quantitative proteomics became technically possible (Anderson and Anderson, 1998; Sperling, 2001) and its combination with genomics was presented as a major solution for better understanding human biology (Zivy and de Vienne, 2000). This was followed by other -omics technologies, from imaginomics such as documenting circuits relevant for psychiatry (Artigas et al., 2017) and the 100,000 subject UK Biobank imaging project (Miller et al., 2016) over metabolomics (Beger et al., 2016) to lipidomics (Yang and Han, 2016). Together with large cohorts that are followed longitudinally such as Alzheimer’s Disease NeuroImaging project (Weiner et al., 2016), or access to electronic health records, this has generated large databases to the point that the major challenge today is not to generate these data, but to analyze them properly and develop actionable predictive platforms. Such analytical approaches include pathway enrichment strategies and mapping the genetic information onto protein–protein interactions network.

Artificial Intelligence (AI) and Deep Machine Learning are the latest developments in the analytical toolbox to cope with this enormous amount of data (Mak and Pichika, 2018) and they have been quite successfull in analyzing imaging data (Gao et al., 2018), in identifying new chemical entities for drug discovery (Besnard et al., 2012) and in drug repurposing (Gunther et al., 2003). However, for predictive outcomes useful in drug discovery for new targets, the associative nature and the lack of transparency of the “black box” still necessitates the intervention of domain experts to make sense of the predicted outcomes. For example, experimental studies and domain expertise was used together with machine learning and information theory in a rational polypharmacology study of new targets in axon growth (Al-Ali et al., 2015).

As illustrated below, we believe that the combination of “Big Data” with AI analytical techniques together with the “Smart Data” approach of formalizing domain expertise in a Quantitative Systems Pharmacology approach represent a powerful approach for addressing the deadlock in the development of successful CNS therapeutics.

## The Pendulum Swings Back: 21Th Century Target-Agnostic Drug Discovery

The previous sections highlight the challenges of rational and selective target-driven CNS drug discovery. Many of these approaches have also relied on the use of traditional animal models to assess potential clinical efficacy, approaches that also have been questioned as reliable indicators of clinical translation. To address the issue of effective translatability, new technologies have been developed that allow researchers to humanize preclinical models, including human IPSC cells, brain organoids and transgene primates (see a recent workshop from the National Academy of Sciences^1^). However, these approaches, though promising, are still in development and each of them has specific issues. For instance, hIPSC cells, while important for elucidating neuronal biology, lack the specific neuronal circuitry of the human brain. Brain organoids are notably difficult to standardize and have extremely low throughput. Transgene primates have a very low capacity and extremely long timelines.

More importantly, imaging studies, have led to a growing realization that modulating neuronal circuits rather than specific molecular targets can affect clinical evaluation scales considerably. It has indeed become increasingly clear that complex and “abstract” human mood states can be traced back to brain network dysfunction. For example, a triple network model of autism in humans (Menon, 2011) proposes that aberrant functional organization of the Salience Network (SN), the Frontoparietal (FPN), and Default Mode Network (DMN) is related to clinically relevant brain states, such as lack of accurate self-appraisal (Hogeveen et al., 2018). Examples in other indications suggest that drug interventions can affect brain networks. For example nicotine (Faulkner et al., 2018), ondansetron (Stern et al., 2019), and an experimental a7 nAChR agonist (Barch et al., 2016) illustrate the possibility of using this modality for detecting functional network engagement in a clinical trial.

An interesting development has been the introduction and development of the “SmartCube” approach where high-dimensional behavioral data are automatically captured in animals after therapeutic interventions (Alexandrov et al., 2015). The “signature” produced after the administration of a novel experimental drug can be compared to the relevant signature of several clinically active “reference compounds.” One can then use this approach to optimize a compound with “antipsychotic” or “antidepressant” properties based on the specific behavior as analyzed by the Smart Cube in a preclinical setting. Such a strategy is only possible in animal models within a specific genetic strain and in highly standardized and automated conditions to significantly reduce the variability and reproducibly detect the pattern associated with the drug pharmacology. One such example to emerge from this approach is SEP-363856 currently in clinical development for schizophrenia and psychosis in Parkinson’s Disease^2^. It will be interesting to see whether this approach translates into the more variable human patient population.

Techniques such as optogenetics (Zhang and Cohen, 2017) and DREADD (Dobrzanski and Kossut, 2017) allow for the manipulation of neuronal circuit activity and relate these to behavioral outcomes in animal models. These interventions are currently off-limit for use in human patient populations, but repetitive Transcranial Magnetic Stimulation and direct current stimulation approaches on the human brain can provide insights in neuronal activity and its relation to clinical outcomes.

### The Case of Ketamine as a New Antidepressant

The potent anti-depressant effect of low-dose iv Ketamine [introduced as an anesthetic in the 1960s (Domino et al., 1965)] was identified in a clinical trial (Zarate et al., 2006a), while another NMDA modulator, memantine was without effect (Zarate et al., 2006b). 10 years later the exact mode of action has not been identified although effects beyond NMDA-R modulation are considered as a prime candidate (Williams and Schatzberg, 2016), but also Homer1A modulation (Serchov et al., 2016) and the mTOR pathway (Ignacio et al., 2016) have been proposed. As a consequence, a number of companies are developing products based on new formulations (e.g., intranasal administratio of esketamine by Janssen), and analogs of ketamine or drugs based on the NMDA modulation properties (Biohaven Pharma, Mnemosyne, Naurex, Neuropinc). Future clinical trials will reveal whether the NMDA pathway is a major driver of the anti-depressant effect. This is another example where smart clinical observations led to the discovery of a whole new pathway to treat major depression, essentially in the absence of a rational theory. Indeed, the FDA recently approved the use of esketamine (Spravato^TM^ ) for use in treatment resistant depression.

## Quantitative Systems Pharmacology as a Phenotypic Assay

**FIGURE 2:**
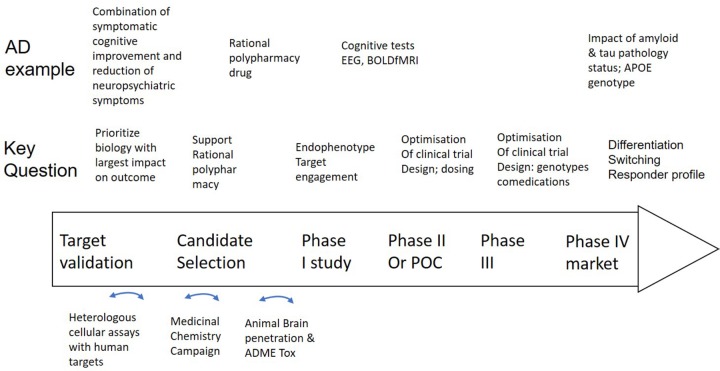
Integration of a mechanism-based QSP model in CNS drug discovery and development projects. Well validated QSP models could help not only in identifying, but also validating new and powerful targets that can restore neuronal circuit dysfunction, can support rationally designed MedChem campaigns and help select the best clinical candidate (middle row). Experimental work (bottom row) includes testing of drugs in heterologous cell systems, execution of a MedChem campaign with a focus on synthesizing multi-target compounds and the study of brain penetration and ADME-Tox in preclinical animal models. Clinical trial design (middle row) can be supported by Hyman (2014a) identify the optimal dose, (Geerts, 2009) explore the effect of the drug on non-invasive biomarkers and (Hyman, 2008) identify negative pharmacodynamic interactions between a novel drug and comedications, genotypes and disease states by simulating virtual patients. In the case of AD as an example (top row), such a project can be applied to the development of a drug that addresses both symptomatic cognitive and neuropsychiatric symptoms, followed by brain region activity related biomarkers, such as EEG and BOLDfMRI and the impact of tau, amyloid status, comedications, and APOE genotype on the dose-response of the new investigative drug.

We propose that Quantitative Systems Pharmacology (QSP), based on complex and clinically calibrated computer models of human neuronal circuits, is a major new tool that can also incorporate the new experimental technologies in development mentioned above. Technical descriptions of the QSP platform have been published extensively (Spiros et al., 2010; Geerts et al., 2012, 2013b), here we only give an overview of the QSP approach with a focus on applications along the drug discovery spectrum (see Figure 2).

Basically, the platform simulates the firing characteristics of a biophysically realistic neuronal network that integrates physico-chemical and physiological data from preclinical models and imaging, functional genomics and postmortem data in patients. The platform uses biophysical Hodgkin-Huxley membrane potential calculations of neuronal networks that are modulated by different neurotransmitter circuits. This allows representations of targets of approved CNS active medications. Common functional genotypes can be implemented using human imaging studies (Spiros and Geerts, 2012). The pathological state is introduced using imaging and biomarker studies that differentiate patients from healthy subjects. This allows the approach to go beyond the pathology implementation in preclinical animal models where usually only one aspect is modeled, for instance with transgene animals. The model is further calibrated by simulating all currently available historical trials with a large number of drugs and optimizing the outcome with actual reported clinical values. A receptor competition model simulates the competition between therapeutic agents and endogenous neurotransmitters based on their affinity to pre- and post-synaptic receptors and integrated with a model of presynaptic physiology on facilitation/depression of synaptic vesicle release and the negative feedback of presynaptic autoreceptors (Spiros et al., 2010). This module allows for the calculation of the functional free intrasynaptic active drug moiety concentrations derived from PET imaging studies by adjusting the concentration of drugs to match the observed displacement of the PET tracer. The changes in activation level of the postsynaptic receptors are then translated to the appropriate changes in voltage-gated ion channel conductance by optimizing the correlation of the model output with clinical data. In some cases, the platform has been able to make prospective blinded and correct predictions on clinical trial outcomes.

The application of QSP in drug discovery will be elaborated in detail in the following section, but essentially would start from reverse engineering the platform to identify a validated “lean” target product profile for a medicinal chemistry campaign. Molecular Modeling and other Big Data approaches could be used for accelerating and supporting the MedChem part. Assays of heterologous *in vitro* cellular systems with these human targets will be developed as a first-line screening of candidate drugs with a fast feedback cycle for a new round of medicinal chemistry. The most promising candidates will be tested in a more extensive screening against a large number of additional targets to complete its full pharmacological profile.

The QSP-based strategy would essentially complement the *in vivo* pre-clinical efficacy models for driving Go/NoGo decisions by “extrapolating” the often substantial animal pathology to the human situation in an appropriate spatio-temporal context. However, animal models would still be needed to confirm central target engagement and mandatory ADME-Tox related studies and in some cases to study the pharmacodynamic effect of the selected dug candidate in a “relevant” disease model. In any case, this could substantially reduce the need for animal studies, speed up the drug discovery process and reduce costs.

It is worthwhile considering the fundamental difference between this domain-expertise based approach and the data-driven Artificial Intelligence/Machine Learning techniques (AI/ML) (Geerts et al., 2016a, 2017). Machine learning approaches are very good for classification purposes and pattern recognition in large datasets, but they need high-quality training sets and do not include previous domain knowledge. For new targets in drug discovery, where there are few or no data available and pharma companies learn on the “fly,” they are less useful. The algorithms are basically a black box and are not based on biological understanding. Moreover, at best they can derive correlations, but lack the capability to address very concrete questions such as “how much do I need to modulate that pathway to have an appropriate balance between efficacy and side-effects?” or “what is the optimal dose for my drug that affects this new target?”

In contrast, because QSP is based on formalized and quantitative domain expertise and knowledge about fundamental biological processes, it can more easily predict outcomes without the need for “training” sets that include these novel targets and therefore is more readily generalizable, compared to AI processes. For instance, the QSP platform blindly and correctly predicted an unexpected clinical outcome for a pro-cognitive 5-HT4 drug acting on a novel target and was able to identify the translational disconnect between human subjects and rodent models (Timothy et al., 2013) in the absence of any clinical “training” data on this novel target. At the same time, simulations showed that the back-up compound with a different pharmacological profile would have a better clinical outcome. It is conceivable that future developments of AI and ML could combine biological insights captured in publications through natural language processing with clinical data from electronic health records to generate predictions of as yet untested clinical targets.

### Preclinical Applications

#### Validation of Targets

Quantitative Systems Pharmacology allows not only for the possible identification of a new target product profile but also for a certain degree of validation that predominantly drives a specific clinical scale outcome to be used in drug discovery through “reverse engineering.” Within a well validated computer model, a systematic search of all the biological processes and their contribution to a change in phenotype (for example reversal of the clinically calibrated emergent properties from a pathological to a normal state), -essentially a sensitivity analysis – can identify key pathways and targets for novel drugs. For example, using this approach (Geerts et al., 2015b) the top biological processes that characterized responders to iloperidone, a recently approved antipsychotic drug, were found to be the coupling factor between cortical D4-R and the AMPA receptor, in line with a SNP found in the GRIA4 gene in a traditional PGX analysis (Lavedan et al., 2008). Along the same lines, an extensive analysis of all combinations of biological processes in the model can possibly identify synergism of a polypharmacy profile.

Another example illustrates the capability of QSP to identify a novel neuronal circuit that can drive a more complex clinical phenotype such as psychosis. Using an advanced computer model of a closed cortico-striatal-thalamocortical loop (Spiros et al., 2017) with schizophrenia pathology derived from human imaging studies, the changes in firing dynamics in the Thalamic Reticular Nucleus correlated best with the clinical antipsychotic effect of a large number of drugs. This area is strategically located between thalamus and cortex, controls the information flow at this critical juncture of the closed loop, and has been identified as a critical neuronal endophenotype in neurodevelopmental disorders (Krol et al., 2018). Recent studies using EEG/MEG have identified this area as crucial for spindle mechanisms (Piantoni et al., 2016) that are dysfunctional in schizophrenia. In principle this could lead not only to the identification of novel targets, but also their validation in a neuronal circuit that is essential for psychiatric symptoms.

A substantial limitation of our QSP approach, however, is the restriction to targets for which there is sufficient biological knowledge available. While sometimes modern drug discovery projects start with a limited amount of knowledge (usually a correlation based on genetic information), a mechanism-based QSP approach needs more elaborate biological data on the cell type, specific neuronal circuit, intracellular pathway or substrates. This limits the space of possible targets but has the advantage that the selected targets have a much broader biological knowledge and possibly a higher chance for success.

#### Generating Actionable Knowledge From Preclinical hIPSC Experiments

In order to mitigate the translational disconnect between rodent preclinical models and the human patient, neuronally differentiated hIPSC or brain organoids have become increasingly popular as tools for drug discovery. As an example, using advanced transcriptomic analysis of drug effects on human derived hIPSC cells, a number of drugs that reversed a “schizophrenic” signature back to normal was identified allowing for the exploration of entirely new pathways for treatment (Readhead et al., 2018).

**FIGURE 3:**
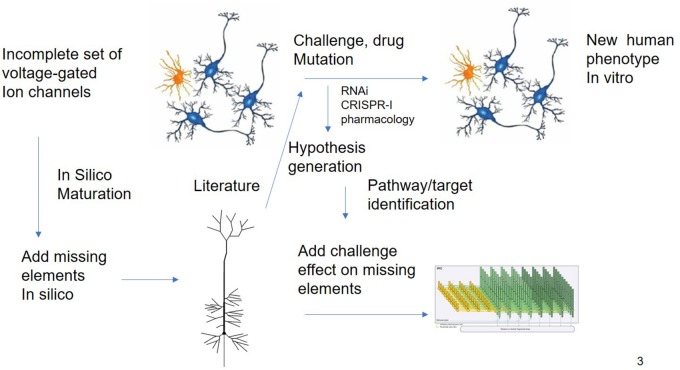
*In silico* Translation of hIPSC Cell Culture Experiments to *in vivo* “Humanized” Situation. Experiments using different modalities (electrophysiology, biochemical studies, and transcriptomics) are limited by the issues on differentiation status. QSP could in principle extrapolate the findings of these experiments by “adding” missing key biological processes present in human cells. The interpretation of challenge experiments (pharmacological or genetical) could be enhanced by knowledge driven modeling of the underlying biology. Finally, these findings could be integrated into a neuronal circuit model that is closer to the clinical scales.

While this example is based essentially on transcriptomic readouts, other more functional experimental readouts such as electrophysiological studies on Multi-electrode Arrays (MEA) can report on more subtle deficits and are more amenable to mechanism-based simulations. The effect of therapeutic interventions on neural progenitor cells from patients or the change in electrophysiological properties due to the disease can be studied using multi-electrode arrays (Hondebrink et al., 2017). In principle, an *in silico* model of such an experimental set-up can be developed to perform a systematic search of modifications of voltage-gated ion channels for reproduction of the experimental phenotype, prioritizing a list of possible hypotheses that can be tested experimentally. This could lead to novel targets and biological insights (Figure 3).

#### Computer Assisted Synthesis of Rational Polypharmacy

Starting from the ‘ideal” lean profile described above, different approaches can then be used to generate chemical structures with the desired polypharmacy. One such approach relies on identifying chemical structures that act on individual targets using searches in chemical databases, followed by finding common pharmacophores of molecules acting on the different targets and developing a chemical “template” that can serve as a start for a medicinal chemistry campaign. Other approaches use Big Data analytics and AI or deep learning for predicting chemical structures with a well-defined multi-target profile (Besnard et al., 2012).

Once this ideal profile is established and a medicinal chemistry campaign is started, there is a need for an “assay” that can test profiles of new leads coming out of such a program. Because these compounds are aimed at changing neuronal circuit properties rather than single targets, cell culture models such as hIPSC cannot be used. Rather than using expensive and low throughput animal models, in principle, a QSP platform that is well validated can play a major role as an *in silico* tool to prioritize different candidate leads based on their pharmacological properties derived from individual human target assays (Geerts and Kennis, 2014). This would both accelerate the selection of the best clinical candidate and ensure a higher level of translatability.

As a great application of such a rational polypharmacy approach in Alzheimer’s disease, the strategy to combine a pro-cognitive pharmacology with a disease-modifying target would substantially de-risk clinical development, using short-term (6 months) clinical trials for symptomatic improvement, getting marketing approval and then perform the long-term clinical studies (2–3 years) to confirm the disease-modifying properties in order to change the label.

### Clinical Applications

#### Identifying Biomarkers of Target Engagement in Human Populations

A major challenge in early clinical development is the ability to estimate the degree of functional target engagement in a Phase I study with healthy volunteers. In some cases, PET imaging tracers are developed alongside the therapeutic compound to estimate the degree of receptor occupancy or the target product profile includes a target for which there is already a clinically approved PET tracer available.

If these tracers are unavailable, functional non-invasive biomarkers such as EEG and BOLDfMRI can be used as elaborated above in section “The Pendulum Swings Back : 21th Century Targetagnostic Drug Discovery.” Because these are based on neuronal circuit activity, they fit very well with the concept of QSP modeling. Indeed complex computer models for simulating both resting state (Rosen et al., 2018) and evoked EEG responses have been developed (Moxon et al., 2003) and can be extended to include the effect of pharmacological interventions that affect membrane potential dynamics such as those downstream of membrane G-Protein Coupled Receptors (GPCR) targets. Similarly, voxel-based BOLDfMRI can be simulated from firing activities in neuronal circuits (Sotero and Trujillo-Barreto, 2007). An additional advantage of QSP is that these observations in healthy volunteers can be extrapolated “*in silico*” to a pathological situation.

#### Identifying the Best Patient Population for the Investigative Drug

Because the QSP platform is based on the biology of the human brain, different clinical indications or disease states can be explored to identify the most responsive patient population. For instance, pro-cognitive interventions such as acetylcholinesterase inhibitors could be positioned either for symptomatic relief in dementia (Roberts et al., 2012) or cognitive impairment in schizophrenia (Geerts et al., 2013a, 2015a). In AD these drugs can be tested as the primary intervention, but their efficacy can be dependent upon the β-amyloid load, while in schizophrenia they must be given as augmentation therapy with antipsychotics in order to provide efficacy for the psychotic symptoms. As many antipsychotics have a rich pharmacology, PD–PD interactions can significantly affect clinical outcome as illustrated above in section “Highly Selective Drugs in CNS Disorders.”

In another example, amyloid load at baseline can affect the cognitive readout of amyloid reducing interventions (Geerts et al., 2018b). This is due to the fact that the shorter Aβ forms have a dose-response in which they are predominantly neuro-stimulatory, while the longer Aβ forms reduce glutamate neurotransmission irrespective of the dose. These non-linear effects, first detected in preclinical studies are likely active in human patients as they are essential to explain three different clinical datasets. These simulations suggest that amyloid reducing interventions are only improving cognition for patients with a high amyloid baseline, while they reduce cognitive performance in subjects with low or zero amyloid.

#### Identification of Negative Pharmacodynamic Interactions

In clinical CNS trials as well as in clinical practice, polypharmacy is the rule rather than the exception. For instance, AD patients are often treated with acetylcholinesterase inhibitors and the NMDA-inhibitor memantine, but additional CNS-active co-medications are often added with an estimated 40% taking antidepressants and 20% of AD patients taking antipsychotics for behavioral disturbances such as agitation (Clague et al., 2016). Current guidelines are in place for PK–PK interactions whereby one drug affects the metabolism of the other drugs or where a specific genotype of the metabolizing enzymes determines individual drug dose. However, the often complex pharmacodynamic interactions (PD–PD) with novel treatments are not very well studied, most likely leading to a less than optimal treatment paradigm. As an example, the use of drugs with direct or an indirect anticholinergic activity leads to a higher risk of AD (Gray et al., 2015; Risacher et al., 2016) while polypharmacy is associated with lower cognitive performance (Lau et al., 2011).

Because the QSP platform includes the targets of all CNS active drugs together with a simulation module that – in case of antipsychotics – calculates level of functional target engagement based on PET imaging, the effects of these co-medications on the dose-response of a new investigative drug can be predicted.

Similarly, imaging studies of tracer displacement document the effects of common variants such as COMTVal156Met (Slifstein et al., 2008) or 5-HTTLPR rs23351 (Fisher et al., 2012) on the dynamics of dopamine, norepinephrine and serotonin and can be explicitly modeled as a change in neurotransmitter half-life. These genotypes can affect the dose-response of a new drug on cognitive outcome in complex non-linear of ways. Having this information early on, would allow either to modify the exclusion-inclusion criteria or at least stratify the patients over the different treatment arms, possibly leading to a higher probability of success.

#### Analysis of Clinical Trials at the Single Patient Level

*Post hoc* analyses on failed clinical trials are mostly based on statistical analyses using patient subgroups, often based on discrete features such as age and gender, the number of co-medications, the class co-medications or single genotypes such as APOE. This approach, although necessary for achieving sufficient power, does not take into account individual patient characteristics, for instance, the nature of each co-medication (not all antipsychotics or benzodiazepines are the same) or their dose. In addition, combinations of co-medications (for instance antidepressants with antipsychotics) have often non-linear interactions which are heavily dependent upon the nature and dose of the drugs (Geerts et al., 2018a). Other confounding factors conveniently assumed to have no impact include combinations of genotypes. For instance, there are 27 possible combinations of the three common variants APOE, COMTVal156Met, and 5-HTTLPR rs 23351 which all affect cognitive readout in a different way.

We would argue that developing individual QSP models for each patient (“virtual patients”) with their unique medication and genotype profile is a powerful way to extract more information from these *post hoc* analyses. Even with “failed” trials (as most are), there is always a fraction of “responders” and studies of these individual responses can elucidate specific interactions between the drug and the pathology. This level of granularity allows the identification of subtle differences with often large consequences and can lead to better insights on the interaction of the investigative drug with the unique biology of the patient.

#### Combing Pharmacotherapy With Behavioral Therapy

Finally, the combination of Cognitive behavioral therapy and repetitive transcranial stimulation (rTMS) can be optimized using QSP modeling. Although such an approach is currently being tested in CNS disorders with modest success (Nguyen et al., 2017), challenges remain for identifying the optimal conditions for such combination therapy. It is conceivable that an appropriate synchronized combination with the right pharmacotherapy might be synergistic, because of the possible priming by the drug of relevant intracellular pathways which are used by the behavioral intervention For instance, a mathematical model based on the intracellular activation of an ERK-based and a cGMP-based pathway that affects the glutamatergic synapse, takes into account the temporal relationships between the stimulatory pulses and the “priming” of the intracellular pathways, in this case the increase of cGMP after PDE9 inhibition (Smolen et al., 2014). This model has been shown to predict correctly the change in LTP using different timing of stimulatory pulses in an Aplysia experimental model (Zhang et al., 2011). Pharmacological modulation of G-protein coupled receptor activation levels leading to intracellular second messengers such as cAMP or inhibition of phosphodiesterases can affect downstream proteins such as voltage-gated or ligand-gated ion-channels either directly through phosphorylation or via modification of protein synthesis and therefore modulate the action potential dynamics, increase neuroplasticity and clinical cognitive outcomes. In a certain way, well-defined cognitive stimuli in humans do have similarities to electrical stimulation in preclinical models of long-term potentiation (LTP). As another example, the Reset-O program, a digital therapeutic in combination with standard-of-care drug treatment for substance abuse (Ben-Zeev et al., 2016). Therefore, advanced QSP mechanism-based modeling of humanized circuits can, in principle, optimize the timing of combination therapy based on the PK profile and the effect of a novel drug on the priming of intracellular pathways.

## Disease Modifying Approaches

The discussion so far has been focused on the restoration of neuronal circuit homeostasis to achieve a clinically relevant outcome. However, recent R&D projects for neurodegenerative diseases focus on addressing key pathological processes such as β-amyloid dynamics or tau pathology in AD or α a-synuclein in PD. Despite a strong genetic rationale for some of these targets, there are multiple challenges, as evidenced by the failure of multiple clinical trials mentioned previously.

For instance, the complex biology of the β-amyloid peptide with a neuroprotective non-linear dose-response for shorter forms and a neurotoxic role for longer isoforms (Geerts et al., 2018b), and the differences in amyloid load between preclinical animal models and the human situation are drivers of the translational disconnect. Another challenge refers to the large number of comorbidities converging into the aging brain. A third consideration is the multi-scale nature of the processes together with the time scales involved leading from pathological changes to cognitive outcome which by itself is driven by neuronal firing activities of interacting brain regions. This highlights the difficulty of extrapolating time- and spatial scales from the preclinical animal model to the human patients.

These considerations suggest the need for a broader understanding of these multi-dimensional processes, their relationships, the ability to quantify these properties and their ultimate impact on neuronal activity. Computer modeling can be a tool to address some of these issues.

New implementations of astrocyte and microglia biology can expand the current electrophysiology-based approach for instance by modeling the role of microglia in clearing misfolded amyloid and tau proteins or secreting various cytokines that affect neuronal physiology (Edelstein-keshet and Spiros, 2002). Astrocytes can influence neurotransmitter homeostasis by their effect on uptake and synthesis of key neurotransmitters such as glutamate.

Modeling intracellular processes that regulate post-translational modifications of key proteins, such as the tau protein (Stepanov et al., 2018), needs to be coupled to membrane protein modifications that regulate membrane potential dynamics. Model parameters for instance, can be constrained by comparing anticipated outcomes with “fingerprints” of tau molecules, such as phosphorylation at specific sites, detectable in biofluids. Multi-scale modeling not only involves enzymatic processes but also gene regulation, each with appropriate timing dynamics. While the fundamental biology would be informed by preclinical data in cellular systems and transgene mouse models, the QSP platform can be used to scale the predictions to the human pathology case. This is especially important for both spatial and temporal pathology progression (measured by PET imaging and clinical trajectory). This illustrates the different levels of biomarkers that can be used to constrain and validate the model.

As an example, there is sufficient preclinical data to develop a QSP platform of spatial tau progression by combining secretion processes in the extracellular space, subsequent uptake by an afferent neuron, axonal transport over the neuronal projections and progressive conversion of monomeric tau into larger aggregates. At the same time, the interaction of the modified tau protein and membrane proteins, notably voltage-gated ion channels (Hall et al., 2015), can be simulated leading to changes in membrane excitability, firing dynamics and finally behavioral outcome. Combining these processes together with spatial progression as measured by tau PET imaging ultimately can generate a model that accounts for disease progression and has the capacity to start addressing the variability of the patient populations in their clinical phenotype.

## Discussion and Conclusion

### Drug Discovery Focused on Neuronal Circuitry

This report presents a novel paradigm for drug discovery and development that is based on advanced computer modeling supplemented by insights generated from a limited amount of preclinical studies. Basically, the strategy is to reduce the use of preclinical animal models of efficacy with advanced humanized and empirically based computer modeling with the hope of increasing the probability of clinical success. The approaches outlined in this article would significantly reduce the use of expensive animals and reduce both the timeline and resources of selecting the best clinical candidate.

Although the approach presented here is not a “blind” phenotypic assay as it is based on a complex interacting network of well-known neuronal and biochemical circuits, it enables a search of possible combinations that might significantly synergize to revert the pathological phenotype in a target-agnostic way. In fact, the platform aims to deliberately search for a poly-pharmacological profile with known targets to address different pathological imbalances in the relevant circuits and networks. This obviates the need to start expensive target identification studies in other cellular or animal-based implementations of phenotypic assays and opens new avenues for therapeutic innovation.

A major difference with animal-based drug discovery approach is the translatability of the humanized computer model, as this will be extensively calibrated with historical retrospective data and validated with a different historical clinical dataset. In a few cases, such a QSP model has been able to prospectively and correctly predict clinical outcomes in schizophrenia and AD that was different from the preclinical animal-based predictions. Such a tight relationship is an essential criterium for any phenotypic assay. In addition, the platform makes extensive use of the non-invasive and clinical biomarkers that characterize the pathology or the effects of drugs.

The emphasis of the computer modeling on electrophysiological signatures in neuronal firing networks is in line with an increased focus on symptoms classes (Cohen et al., 2017) that are cross-diagnostic (Kas et al., 2019) and is more closely and mechanistically related to underlying neurobiology processes. The biological processes underlying these symptom classes can be probed by non-invasive methods such as EEG and MRI imaging. The ability of the computer model to simulate these outcomes adds a level of translatability to this new paradigm.

Using a computer model for driving the drug discovery process allows a seamless integration with other modeling approaches used during the development phase that are key to a successful project, such as SimCYP (Physiology-based pharmacokinetic modeling). In fact, such an organic integration allows also for a more powerful calibration and validation phase based on the clinical outcome of individual patients as it takes into account PK profiles and blood-brain transport. The capability of developing individual computer models for patients with different genotypes, co-medications and disease status can be very helpful even in early stages of drug discovery, as it allows for the possible identification of back-up candidates that can be tailored for a different patient population.

### Limitations of This Approach

First and foremost is the limited scope of the model; many more biological processes or brain regions are not included. In contrast, animal models do have a complete brain biology but some of these processes might be irrelevant or very different from the human situation. There is therefore a trade-off to be made between focusing on key human-disease relevant processes versus including biology that might have a limited contribution to the global outcome. In this respect, close inspection of phenotype databases (including biomarkers) can help in prioritizing the key circuits that are driving a clinically relevant outcome (for instance the neurocircuitry of an RDOC dimension). Also, the hope is that these computer models in the future will expand considerably given the availability of increased computer power and storage.

Secondly, the approach relies heavily on the extrapolation of *in vitro* pharmacology using artificial cellular systems to the “humanized” *in vivo* situation. While this is to some degree also an issue for the more traditional animal-based drug discovery process, it can to some extent be mitigated by the use of human patients derived hIPSC cells.

Thirdly, the computer model is based on actual knowledge and cannot account for targets and pathways that are still to be discovered and important for the clinical phenotype. In this regard new findings from large GWAS studies can identify pathways that need to be prioritized for modeling in future iterations.

Fourth and most importantly, the unique validation of this approach relies on the availability of clinical data from past trials, especially on an individual patient basis. Although these datasets become increasingly available for other indications, many companies in the CNS area are reluctant to provide information on their interventional trials. This is a crucial prerequisite and, although some headway has been made using published group average data, as in the current generation of QSP models, there is substantial room for improvement.

We acknowledge that this approach is a fundamental paradigm-shift compared to the more traditional animal-based drug discovery strategy. However, it has become abundantly clear repeated late-stage failures have resulted in increased pressure for innovative approaches. This new paradigm focuses on data and knowledge, not animal models or chemistry, as the most important assets in pharmaceutical discovery and development. Moreover, where there is a noteworthy paucity of drugs to treat neurological disorders, it is possible that the approaches outlined in this article may also extend to drugs effective in treating those conditions as well. A number of start-ups (i.e., Numedii, Benevolent AI, Berg Pharma) use Artificial Intelligence and Deep Learning approaches to identify new targets for drug discovery – although so far not in CNS disorders -; however, they still need to “validate” their targets in animal models as a key transition into clinical trials.

The approach fits into the broader concept of digitizing the pharmaceutical enterprise, spearheaded by technology companies such as Calico and Verily (Google Alphabet), 23andMe and others. We believe that with the right software and modeling approach these companies will ultimately be more successful.

In summary, the advanced computer-based mechanistic modeling as presented here is a powerful tool to integrate a large amount of knowledge (both basic and clinical information) into an actionable platform that provides the opportunity to address key questions along the CNS drug discovery and development spectrum and which can, hopefully, improve the outcome of drugs to treat the spectrum of CNS disorders.

## Data Availability

No datasets were generated or analyzed for this study.

## Author Contributions

All authors listed have made a substantial, direct and intellectual contribution to the work, and approved it for publication.

## Conflict of Interest Statement

The authors declare that the research was conducted in the absence of any commercial or financial relationships that could be construed as a potential conflict of interest.

## Footnotes

1 https://www.nap.edu/catalog/24672/therapeutic-development-in-the-absence-of-predictive-animal-models-of-nervous-system-disorders

2 http://www.sunovion.us/research-and-development/pipeline.html
